# Osteogenic Embryoid Body-Derived Material Induces Bone Formation *In Vivo*

**DOI:** 10.1038/srep09960

**Published:** 2015-05-11

**Authors:** Ken Sutha, Zvi Schwartz, Yun Wang, Sharon Hyzy, Barbara D. Boyan, Todd C. McDevitt

**Affiliations:** 1 Wallace H. Coulter Department of Biomedical Engineering, Georgia Institute of Technology & Emory University, 313 Ferst Drive, Atlanta GA, 30332-0535, USA; 2 Department of Biomedical Engineering, School of Engineering, Virginia Commonwealth University, Richmond, VA, 23284-3068, USA; 3 Parker H. Petit Institute for Bioengineering and Bioscience, Georgia Institute of Technology, 315 Ferst Drive, Atlanta GA, 30332-0532, USA

## Abstract

The progressive loss of endogenous regenerative capacity that accompanies mammalian aging has been attributed at least in part to alterations in the extracellular matrix (ECM) composition of adult tissues. Thus, creation of a more regenerative microenvironment, analogous to embryonic morphogenesis, may be achieved via pluripotent embryonic stem cell (ESC) differentiation and derivation of devitalized materials as an alternative to decellularized adult tissues, such as demineralized bone matrix (DBM). Transplantation of devitalized ESC materials represents a novel approach to promote functional tissue regeneration and reduce the inherent batch-to-batch variability of allograft-derived materials. In this study, the osteoinductivity of embryoid body-derived material (EBM) was compared to DBM in a standard *in vivo* ectopic osteoinduction assay in nude mice. EBM derived from EBs differentiated for 10 days with osteogenic media (+β-glycerophosphate) exhibited similar osteoinductivity to active DBM (osteoinduction score = 2.50 ± 0.27 vs. 2.75 ± 0.16) based on histological scoring, and exceeded inactive DBM (1.13 ± 0.13, p < 0.005). Moreover, EBM stimulated formation of new bone, ossicles, and marrow spaces, similar to active DBM. The potent osteoinductivity of EBM demonstrates that morphogenic factors expressed by ESCs undergoing osteogenic differentiation yield a novel devitalized material capable of stimulating *de novo* bone formation *in vivo*.

Acellular and devitalized tissue therapies have been successfully used to promote endogenous repair to treat a variety of traumatic injuries and degenerative diseases. Tissue derived materials lacking viable cells but comprised of natural extracellular matrix (ECM) components, such as structural adhesive proteins, glycosaminoglycans and bioactive growth factors embedded within the native matrix from the tissue of origin, retain potent bioactivity^1^,^2^. Demineralized bone matrix (DBM) is perhaps the most well-studied devitalized matrix material currently in clinical use due to its well-established capacity to stimulate *de novo* bone formation^3^. Despite widespread clinical use, DBM is subject to several well-known and inherently unavoidable caveats. The *in vivo* osteoinductivity of DBM can vary between bone banks, and from batch-to-batch within the same bone bank, due to varying processing conditions and the heterogeneity of donor tissues^4^. As with any cadaveric-sourced, allograft tissue, limited donor availability and lack of control over donor characteristics, such as age or environmental exposures, can adversely impact the quality and potency (i.e. osteoinductivity) of DBM^5^,^6^,^7^. These limitations of donor tissue-derived therapies motivate the need for reproducible and consistent source material(s) for manufacturing of regenerative acellular products.

Pluripotent cells, traditionally referring to embryonic stem cells (ESCs)^8^,^9^,^10^ and, more recently, induced pluripotent stem cells^11^,^12^, are an attractive source for differentiation of essentially all mammalian cell and tissue types. Pluripotent ESCs, derived from the inner cell mass of blastocyst stage embryos, are capable of extensive self-renewal *in vitro* and differentiation into cell types comprising all three germ lineages (ectoderm, endoderm, and mesoderm)^13^. Due to their pluripotency, these cells are capable of differentiating not only into the osteoblast lineage, but also into associated cell types of the mesoderm lineage, such as endothelial and hematopoietic cells, which contribute to bone formation and maintenance^14^,^15^,^16^. Additionally, ESCs are capable of producing trophic factors that not only regulate adult cell responses *in vitro*^17^ but also support tissue regeneration *in vivo*^18^,^19^, an attribute that is often either severely diminished or completely lost by mature somatic cells.

ESCs can be differentiated as three dimensional cell aggregates, commonly referred to as embryoid bodies (EBs). We have previously derived different forms of devitalized EB materials (EBM) that retain the bioactive factors produced by ESCs undergoing morphogenic differentiation^20^,^21^,^22^. Though similar factors could be delivered from viable transplanted cells, the use of a devitalized ESC therapy, such as EBM, eliminates the risk of teratoma formation associated with the direct delivery of viable ESCs or primitive differentiated cells comprising EBs^8^.

The objective of this study was to determine the osteoinductive potential of a novel material derived from devitalized osteogenic differentiated EBs. As we have previously demonstrated, osteogenic differentiation of ESCs can be stimulated by β-glycerophosphate (βGP) treatment^23^. It was hypothesized that bioactive factors enriched within the embryonic-like microenvironment of EBM, in particular bone morphogenetic proteins (BMPs), would induce bone formation when implanted in vivo. Moreover, it was hypothesized that osteoinductive potential would be enhanced when EBM was derived from EBs undergoing osteogenic differentiation in response to βGP stimulation. As an initial assessment of EBM preparations *in vitro*, the levels of osteoinductive and osteopromotive factors, including bone morphogenetic protein 2 (BMP-2), bone morphogenetic protein 4 (BMP-4), and vascular endothelial growth factor (VEGF) extracted from EBM, were quantified. The osteoinductive potential of EBM was assessed using an *in vivo* mouse intramuscular implantation assay^6^ by quantifying mineralization and the frequency of bone induction, as well as performing histomorphometric measurements of new bone formation in comparison with active and inactive DBM. This proof-of-principle work establishes a novel osteoinductive therapy exploiting the regenerative potential of ESC-derived materials that may be capable of stimulating *de novo* tissue formation for clinical applications aimed at ameliorating tissue injury or degeneration.

## Results

### DBM and EBM Characterization

**Figure 1:**
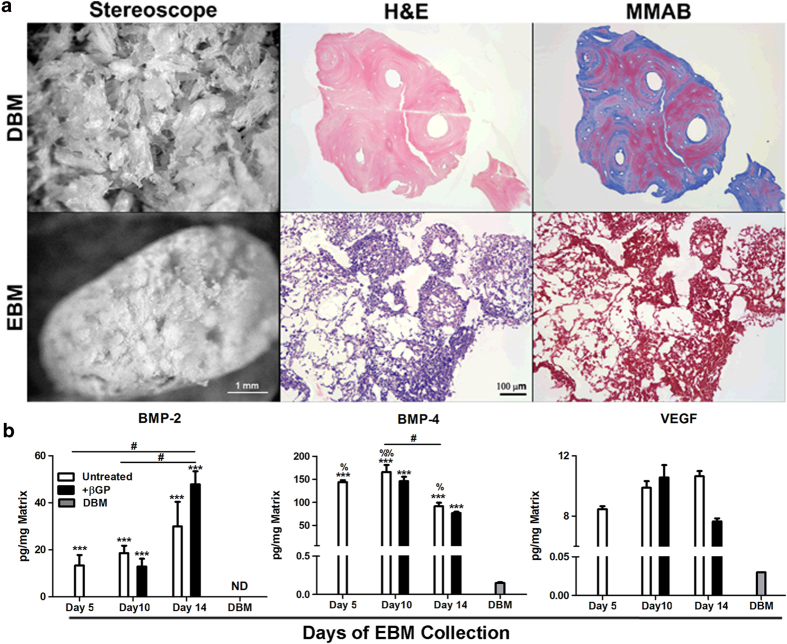
Characterization of DBM and EBM. DBM and day 10 EBM structure and composition were examined macroscopically (left panel, scale bar = 1 mm) and microscopically (center panel: H&E and right panel: modified MMAB, scale bar = 100 μm) to reveal their material architectures and collagen content (**a**). EBM derived from EBs without or with βGP-treatment started on day 5 of EB formation was harvested at D5, D10, and D14. Growth factors extracted from both EBM and DBM were quantified by ELISA (**b**). The error bars represent standard error of the mean. n = 3 samples, Box-Cox transformation, ANOVA, Tukey’s post-hoc test, ***p < 0.005 compared to DBM, #p < 0.05 for marked comparison, %p < 0.05, %%p <0.01 compared to Day 14 +βGP EBM. ND: not detectable.

DBM and EBM were characterized prior to implantation based upon general structure, composition, and extractable growth factor content. DBM was macroscopically identifiable as a hard, dense, and particulate material, comprised of distinct granular pieces with rough edges. In contrast, day 10 EBM exhibited a loosely packed “cotton ball” appearance with handling characteristics similar to that of a dry powder and retained the ultrastructure of individual EBs (Fig. 1a, left panel). The differences in material architecture between DBM and EBM were also observed at the microscopic level, as revealed by haematoxylin and eosin (H&E) staining. DBM was comprised of solid, eosinophilic particles in the size range of 400–1000 μm, whereas EBM exhibited a less dense structure, including some remnant nuclear material, with architecture similar to that of intact individual EBs (Fig. 1a center panel). Analine blue staining revealed abundant, positive collagen staining (blue) of DBM (Fig. 1a, right panel) whereas EBM exhibited little to no collagen content. In addition, EBM macro- and microscopic structures and histological staining were similar regardless of the day of isolation and βGP treatment, consistent with previously published results^22^.

EBM derived from EBs without or with βGP-treatment starting on day 5 of EB formation was harvested at D5, D10, and D14. Several key growth factors important in bone development and repair, including BMP-2, BMP-4, and VEGF were readily extracted from EBM with Tissue Protein Extraction Reagent (T-PER, Pierce). The quantities of extractable BMP-2, BMP-4, and VEGF obtained from both untreated EBM and βGP-treated EBM were significantly higher than samples extracted from DBM using T-PER (Fig. 1b, P = 0.000 for all the comparisons). Osteogenic differentiation of EBs with βGP treatment did not significantly alter the growth factor quantities extracted from day 10 and day 14 EBM; however, the production of BMP-2 and BMP-4 was temporally regulated during the course of EB differentiation. More BMP-2 was extracted from day 14 +βGP EBM compared to day 5 untreated EBM (P = 0.043) and day 10 +βGP EBM (P = 0.026), while BMP-4 content extracted from +βGP EBM was less at day 14 (P = 0.033 vs day 5 and P = 0.03 vs day 10, respectively). VEGF content did not vary significantly during the course of differentiation.

While EBM derived from day 10 and day 14 EBs retained comparable amounts of osteoinductive and osteogenic growth factors, day 14 EBs displayed more endogenous mineralization than day 10 EBs, as evident by more intensive von Kossa and alizarin red staining (Supplementary Figure S1). Therefore, in order to benefit from the morphogen content retained within EBM while minimizing potential interference of visible mineral deposits present in day 14 EBM during *in vivo* mineralization analysis at the site of implantation, EBM derived from day 10 of EB culture was chosen for subsequent *in vivo* evaluation.

### *In Vivo* Mineralization Analysis

Osteoinductivity was evaluated by implantation into the gastrocnemius muscle of the hindlimb of nude athymic mice, a common model for the evaluation of DBM potency^24^,^25^,^26^ that is accepted as the ASTM standard for the evaluation of osteoinductive materials^6^. Heat-inactivated DBM was used as a delivery vehicle for EBM; thus only half the quantity of EBM was implanted compared to DBM (per mass basis) in order to implant a total of 15 mg of materials in each experimental group.

**Figure 2:**
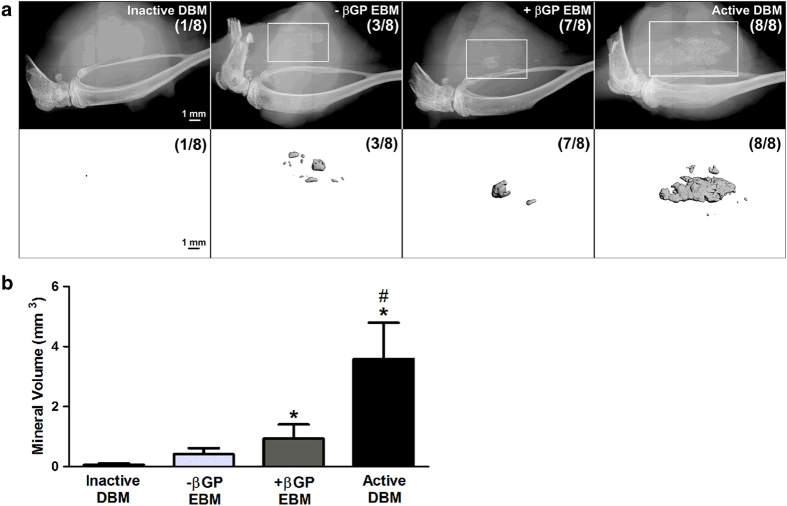
Evaluation of mineralization *in vivo*. DBM and EBM formulations were evaluated in a mouse intramuscular osteoinduction model. Mineralization associated with the material implant sites was visualized by X-ray and the # of limbs with mineralization detectable by x-ray/total number of limbs per group was reported. (**a**, top). Mineralization was further visualized by μCT and the # of limbs with mineralization detectable by μCT (mineral volume > 0.05% of total limb volume [0.10 mm^3^])/total number of limbs per group was reported (**a**, bottom). The total mineral volume per limb was quantified by μCT (**b**). The error bars represent standard error. n = 8 samples, Box-Cox transformation, ANOVA, Tukey’s post-hoc test, *p < 0.05 compared to inactive DBM alone, #p < 0.05 compared to –βGP EBM.

Mineralization associated with the DBM and EBM implant sites was detectable by both X-ray (Fig. 2a, top panel) and μCT (Fig. 2a, bottom panel) 35 days post implantation. Multiple areas of mineralization were present around the site of implantation in all of the hindlimbs implanted with active DBM. In contrast, fewer but more concentrated areas of mineralization were observed in EBM implanted animals, with 7 out of 8 limbs implanted with +βGP EBM exhibiting mineralization compared to only 3 out of 8 limbs and 1 out of 8 limbs implanted with -βGP EBM and inactive DBM, respectively. Consistent with X-ray results, μCT quantification of ectopic mineralization in the same limbs showed a significantly higher volume of mineralized tissue with implantation of both +βGP EBM (0.93 ± 0.47 mm^3^, P = 0.022) and active DBM (3.59 ± 1.20 mm^3^, P = 0.000) compared to inactive DBM alone (0.06 ± 0.04 mm^3^) (Fig. 2b). In contrast, the mineralized volume in the –βGP EBM group (0.41 ± 0.19 mm^3^) was similar to that of the inactive DBM group (P = 0.619), and was significantly lower than that of the active DBM group (P = 0.002).

### Histological Assessments and New Bone Formation

**Figure 3:**
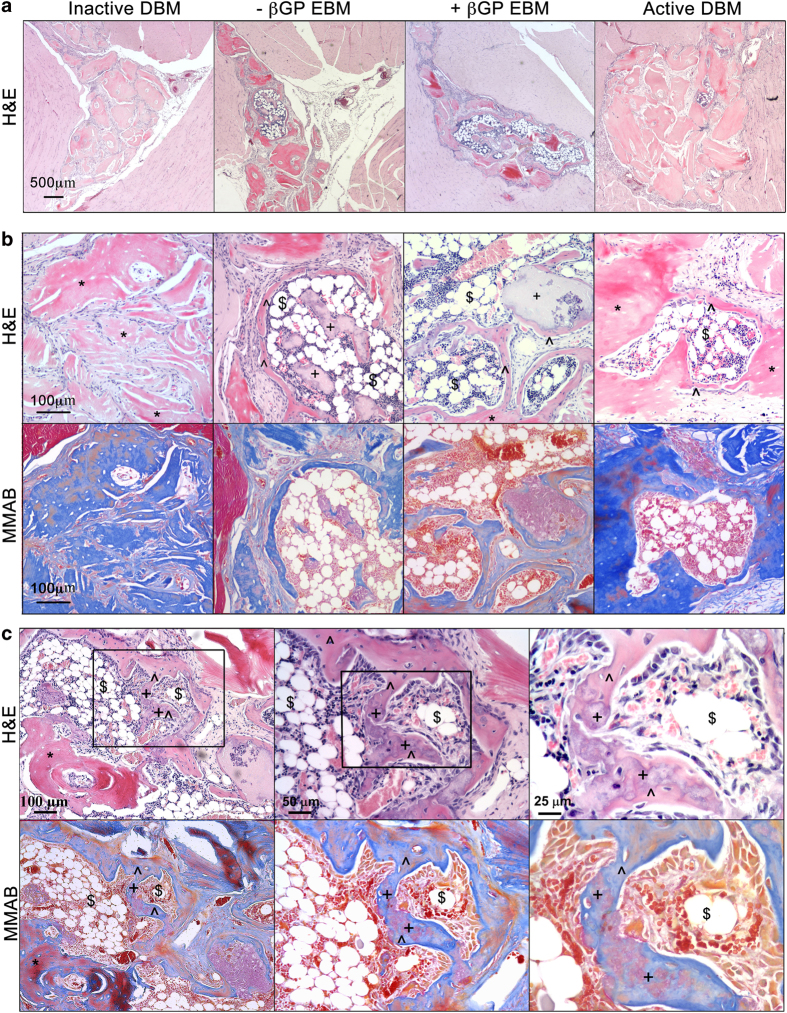
Evaluation of ossicle formation *in vivo*. H&E staining of representative sections from each implant group (**a**: low magnification, 2x and top panel in **b**: high magnification, 20x) and modified Mallory aniline blue (MMAB) staining of adjacent sections (bottom panel in **b**: low magnification, 20x) were used to identify residual DBM and EBM. Higher magnification images (**c**, 40x) of MMAB stained section from **a** +βGP EBM implanted limb was used to demonstrate the new bone formation pattern in +βGP EBM-implanted group. Residual DBM (*), new bone (^), marrow space ($), and residual EBM (+).

The persistence of residual implanted materials within the gastrocnemius muscle was examined following tissue retrieval and histological sectioning. As shown in Fig. 3, consistent with their distinct histological appearance by *in vitro* characterization (Fig. 1a), the remaining implanted DBM (inactive and active) appeared as a dense pink structure by H&E and stained positively (blue) for collagen content by MMAB. In contrast, residual EBM was readily distinguished from co-delivered inactive DBM based upon its looser structure and lack of blue staining for collagen.

The formation of ossicles, characterized by the presence of new bone, the interface at which the amalgamation of new bone with residual implanted material, and the formation of associated new marrow space were also evaluated by H&E stained sections from each implant site. Limbs from both EBM implanted groups exhibited a single large ossicle per section, as much as three times larger than the total ossicle area observed in any of the active DBM-implanted samples. The residual EBM was often localized within or immediately adjacent to the largest ossicles and was also embedded in newly formed bone constituting the large ossicles. In contrast, active DBM implanted samples evoked multiple smaller ossicles, located within a large region of residual DBM (Fig. 3a).

New bone was distinguished from residual implanted EBM and DBM by the distinct mottled staining pattern evidenced via H&E staining (Fig. 3b, top). MMAB analysis confirmed these results, exhibiting dark blue staining for collagen deposition, indicative of bone formation, surrounding areas consistent with mottled regions (Fig. 3b, bottom). New bone integrated along the EBM boundaries in direct apposition to the devitalized material (Fig. 3c), similar in appearance to what was observed at the interface of new bone and active DBM. In many cases, newly deposited bone matrix appeared to be amalgamated with residual EBM based upon positive Analine blue staining engulfing the red-stained acellular material.

### Osteoinductivity Scoring

**Figure 4:**
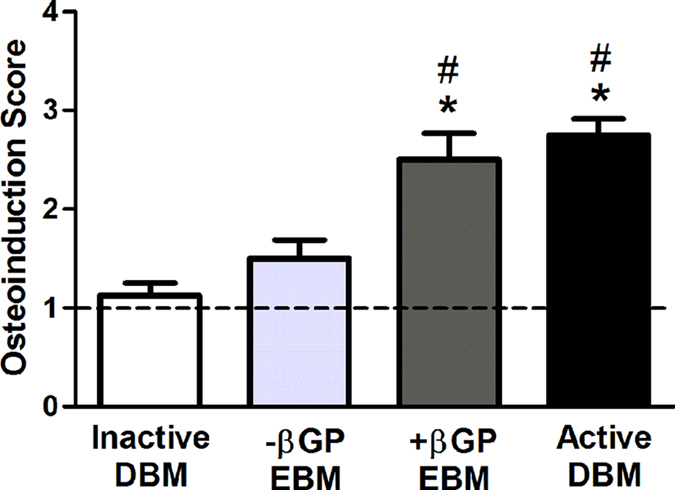
Osteoinduction scoring. Osteoinduction scores were calculated based on a semi-quantitative assessment of ossicle formation in the H&E stained sections. The error bars represent standard error of the mean. n = 8 samples. ANOVA, Tukey’s post-hoc test, *p < 0.05 compared to inactive DBM alone, #p < 0.05 compared to –βGP EBM.

Semi-quantitative scoring of ossicle formation was performed to compare the osteoinductivity of each experimental group (Fig. 4). Both +βGP EBM (score = 2.50 ± 0.27) and active DBM (score = 2.75 ± 0.16) groups had higher osteoinduction (OI) scores compared to the inactive DBM negative control (score = 1.13 ± 0.13, P = 0.00 and P = 0.00, respectively) and -βGP EBM (1.5 ± 0.19, P = 0.01 and P = 0.00, respectively). Moreover, the OI scores of +βGP EBM and active DBM were not statistically different (P = 0.80).

### Histomorphometric Analysis of New Bone

**Figure 5:**
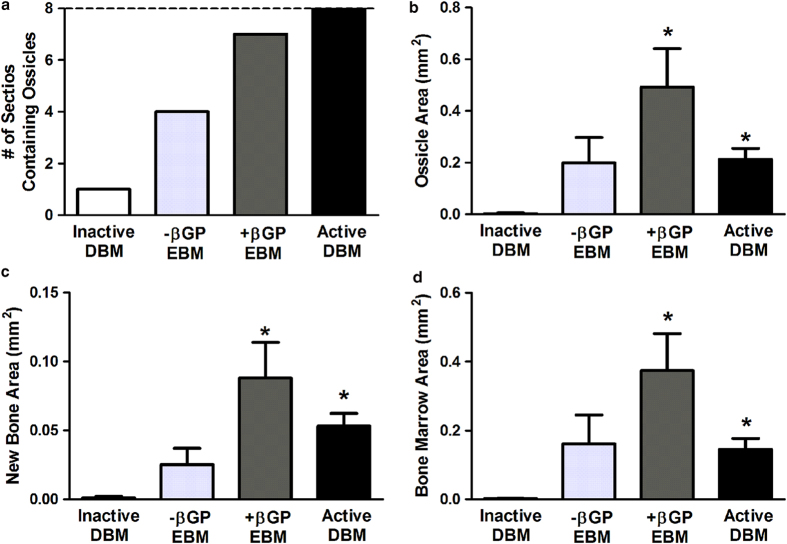
Quantitative histomorphometry. A representative H&E-stained section per animal was evaluated for osteoinduction scoring and histomorphometric measurements, for a total of n = 8 sections (dotted line in a) per group. The number of ossicles (**a**), total ossicle area (**b**), new bone area (**c**), and new marrow space area (**d**) were quantified for each implant group. Error bars represent standard error of the mean. n = 8 samples, Kruskal-Wallis non-parametric ANOVA, Tukey’s post-hoc test, *p <0.05 compared to inactive DBM.

Histomorphometric characterization of the extent of osteoinduction and the composition of new bone was consistent with the overall OI scoring results. Ossicle formation was frequently detected in both active DBM and +βGP EBM groups (Fig. 5a). Ossicle area, as well as the area of new bone and marrow space were all larger with +βGP EBM implants compared to inactive DBM (P = 0.007, P = 0.005, and P = 0.006, respectively) and similar to measures observed in the active DBM-implanted gastrocnemius muscles (Fig. 5b-d, P = 1.000, P = 0.999, and P = 0.999, respectively). Additionally, the histomorphometric values of the -βGP EBM group were comparable to those of inactive DBM (P = 0.770, P = 0.602, and P = 0.758) and were consistently less (but not statistically significant) than those of +βGP EBM (P = 0.063, P = 0.097, and P = 0.064, respectively) and active DBM groups (P = 0.062, P = 0.097, and P = 0.064, respectively).

## Discussion

In the current study, we demonstrate for the first time that ESC-derived materials contain osteoinductive and osteogenic factors (BMP-2, BMP-4, and VEGF) capable of inducing new bone formation *in vivo*. The osteoinductivity of +βGP EBM derived from osteogenic differentiated EBs was comparable to that of active DBM, a commercially available and widely used osteoinductive material, by measures of mineralization, histologic osteoinduction score, and quantitative histomorphometry. Interestingly, the similar osteoinductivity of EBM to that of active DBM was attained despite the delivery of half the amount of EBM, suggesting a greater osteoinductive potency of EBM over DBM (on a per mass basis), which may be attributed to the higher content and accessibility of osteogenic growth factors retained in EBM. Of note, no teratoma formation was observed with EBM implantation within the duration of this study. Thus, the current proof-of-concept study establishes an effective and safe strategy for devitalized pluripotent stem cell-based tissue specific morphogenic therapies.

Pluripotent stem cells have shown much therapeutic promise because of the inherent regenerative potential of immature, less differentiated embryonic environments^27^,^28^; however, the clinical use of ESC-derived therapies has thus far been limited by safety concerns, such as the potential risk of teratoma formation upon implantation if efficient differentiation is not achieved^29^,^30^. In fact, when equal numbers of viable day 10 EBs, either with or without βGP treatment, were implanted in parallel with the devitalized samples described here, teratoma formation was readily observed by 28 days post-implantation, as demonstrated in supplementary Figure S2. In contrast, no teratomas formed within 35 days of implantation of EBM, which can likely be attributed to the complete abrogation of cell viability during the devitalization process. Therefore, the delivery of ESC-derived morphogenic molecules and growth factors represents a safe alternative to harness the therapeutic benefits of the regenerative, embryonic-like microenvironment created by ESCs for *in vivo* applications.

Demineralized bone matrix (DBM) is a natural material capable of harboring osteoinductive and osteogenic growth factors, and remains a leading molecular therapy to induce bone formation. The osteoinductivity of DBM has long been known to be due in large part to its BMP-2 content^3^,^31^,^32^. BMP^-^2 is osteoinductive, and as such, recombinant human BMP-2 is employed clinically as a single factor therapy to promote bone growth and regeneration^5^. In addition to being directly osteoinductive^33^, BMP-4 is critical during early stages of development, including gastrulation and mesoderm formation^34^ and therefore may prime endogenous host cell populations to become responsive to osteoinductive stimuli^35^,^36^. DBM also contains other osteopromotive factors such as the angiogenic growth factor VEGF, which is vital to bone formation, as it promotes neovascularization allowing for the recruitment of the mesenchymal progenitor cells that are induced by BMP-2 to form new bone^37^,^38^,^39^. However, the osteoinductive bioactivity of DBM is confounded by significant donor and lot variability^5^,^7^,^40^,^41^.

Previous studies have measured highly variable quantities of osteoinductive factors within DBM, in both a bone bank-to-bank and batch-to-batch manner^5^,^42^. Measured levels of BMP-2, BMP-4 and VEGF range from 2-120 pg/mg, 0.04-0.4 pg/μg and 0-8 pg/mg DBM^5^,^42^,^43^, respectively. In this study, the quantities of morphogens extracted from DBM were much lower than what have been reported in the literature because a mild extraction reagent (T-PER) was used in lieu of 4 M guanidine-HCl. The actual content of osteoinductive morphogens including BMP-2 present in the DBM used in the present study was likely much larger than what was extracted with T-PER, explaining its greater in vivo osteoinductivity. It should be noted that the positive and negative control DBM batches we used were previously validated using the ASTM standard^6^, supporting our present in vivo observations.

Importantly, employing a relatively mild extraction reagent, we were able to readily obtain osteoinductive and osteopromotive factors from devitalized EBM that are essential for regulating bone formation. More importantly, the quantity of these factors extracted from EBM was significantly higher than that from DBM, suggesting greater accessibility of these morphogens in EBM than in DBM. Additionally, the growth factor content of each batch of EBM was very consistent and much more so than different lots of DBM. Our defined EB culture system with uniform sized EBs and standardized EB culture platform^44^, along with the simple formulation of osteogenic media (βGP only)^23^ successfully minimized batch-to-batch variation and yielded consistent growth factor content of different EBM batches – much more so than independent lots of DBM.

In preliminary studies, EBM alone was delivered *in vivo* to examine its osteoinductivity. However, the inability to identify sites of implantation a month later made it technically challenging to perform ectopic osteoinductivity analyses. Therefore, inactive DBM was used as a vehicle for *in vivo* delivery of EBM in the present study to facilitate the localization of implants due to the unique histological structure of DBM. Although mixing the EBM with inactive DBM readily enabled subsequent analyses, it admittedly complicates the interpretation of the results. Thus, it remains possible that EBM activity is impacted by the presence of inactive DBM. However, the lack of osteoinductivity of HI-DBM alone, strongly suggests that the ectopic bone formation observed in EBM-treated groups is due to the osteoinductive properties of EBM. Future studies of bone repair both with and without inactive DBM or other materials (e.g. collagen scaffold or gel) should elucidate any potential consequences of mixing the EBM with carrier materials.

Despite the fact that only half the quantity of EBM was implanted compared to DBM (per mass basis), comparable osteoinduction scores and mineralized tissue amounts were achieved. Moreoever, histomorphometric analysis revealed distinct differences with respect to the distribution, localization, and composition of newly formed ossicles and associated mineralization in response to implanted EBM. Within both EBM groups, new bone and ossicle formation were observed in sections from all regions in which mineralization was visualized by X-ray, whereas in contrast, regions of mineralization in DBM samples did not always correspond directly with ossicle formation. Additionally, in both EBM groups, new bone tissue usually formed one large ossicle and consisted of a larger marrow space, compared to multiple smaller ossicles induced by active DBM. The increased availability of BMP-2 and BMP-4 in EBM compared to DBM may have yielded the differences in ossicle morphology observed in EBM-implanted muscles, whereby a large area of marrow space was surrounded by a thin layer of cortical bone, versus multiple small ossicles induced by DBM. Similar large ossicle formation has previously been observed in response to direct administration of high doses of recombinant BMP-2 within the same intramuscular osteoinduction model employed in this study^31^. The development of marrow space to support the newly formed bone is important for its maintenance, as it provides a pool of mesenchymal and osteoprogenitor cells that support the continued remodeling of the bone; however, the establishment of marrow is inherently dependent upon the formation of new bone^45^,^46^. Ossicle formation with robust marrow space that resulted from EBM implantation may be advantageous for the establishment of extramedullary bone marrow niches, with potential applications for promoting hematopoietic cell engraftment following bone marrow transplantation. Taken together, the current formulation and dosage of EBM was osteoinductive at a level equal to, if not greater than, the active DBM that was used as a positive control for this intramuscular osteoinduction assay.The similar quantity of extractable osteoinductive and osteogenic morphogens from –βGP EBM and +βGP EBM as indicated by ELISA, might explain why similar levels of ectopic bone formation were achieved by these two groups. However, both x-ray and histological analysis indicated a higher frequency of mineral tissue formation for EBM produced with +βGP treatment compared to -βGP EBM, with 7/8 in +βGP EBM group vs. 3/8 in -βGP EBM group exhibiting positive mineralization by x-ray and 7/8 in +βGP EBM group vs. 4/8 in -βGP EBM group containing ossicles. The most striking difference between EBM formulations was that the osteoinductive score of +βGP EBM was significantly greater than that of the -βGP group. These results suggest that molecular composition of the βGP-treated and untreated groups may be related to differences in the osteogenic differentiation of EBs at the time of devitalization (e.g. 10 days in this study). While the growth factors measured in this study including BMP-2 are believed to be the primary osteoinductive morphogens found in EBM, this study can’t exclude that EBM may contain additional morphogens that also contribute to the ectopic bone formation observed *in vivo*.

As demonstrated here, ESC-derived material can yield *de novo* bone formation by inducing local cellular and tissue morphogenic responses with several potential advantages over adult, allograft-derived therapies. Motivated by the current EBM results, stem cell-derived biomaterials are amenable to controlled production through bulk stem cell culture and bioprocessing, removing donor-to-donor variability that frequently hampers current therapies dependent on cadaveric sourced tissues, such as DBM^31^,^47^. *Ex* *vivo* cell manufactured materials could potentially reduce the need for donor allograft tissue, a long-term goal of tissue engineering and regenerative medicine strategies that has yet to be achieved for many tissues. Future studies to systematically vary culture and differentiation parameters can be performed to further improve the osteoinductive potency or to derive alternative forms of EBM intended for the regeneration of other tissues and treatment of degenerative disorders.

In conclusion, EBM derived from osteogenic ESC microenvironments exhibits potent osteoinductivity, based on its ability to stimulate new bone formation in an ASTM standard evaluation model. The results demonstrate that the osteogenic microenvironment created by differentiating ESCs can be transformed into a cell-derived, osteoinductive biomaterial. This novel finding demonstrates that a devitalized, tissue-specific therapy derived from pluripotent ESCs stimulates directed *in vivo* tissue responses, thereby providing a unique platform to directly translate the regenerative potential of ESCs into clinical therapeutics and motivating the development of ESC-derived materials for a broad array of regenerative medicine applications.

## Methods

### Mouse ESC Culture

Undifferentiated mouse ESCs (D3 cell line) were expanded on 0.1% gelatin coated tissue culture dishes in ESC growth medium containing DMEM (Mediatech, Herndon, VA) supplemented with 15% FBS (Hyclone, Logan, UT), 2 mM L-glutamine (Mediatech), 1x non-essential amino acids (Mediatech), 100 U/ml penicillin/ 100 μg/ml streptomyocin/ 0.25 μg/ml amphotericin (GIBCO, Carlsbad, CA), 0.1 mM β-mercaptoethanol (Fisher, Fairlawn, NJ), and 10^3^ U/mL of leukemia inhibitory factor (LIF, Chemicon, Temecula, CA). Media was fully exchanged at least once every 2 days, and cells were passaged using 0.05% trypsin/0.53 mM EDTA every 2-3 days before reaching 70% confluence (GIBCO).

### EB Formation and Osteogenic Differentiation

EBs were formed by forced aggregation using Aggrewell™ inserts^48^ with 1000 cells per EB (Day 0). After 24 hours of microwell culture (Day 1), EBs were transferred to 100 mm bacteriological Petri dishes (~2500 EBs/dish; 10 ml), and maintained on a rotary orbital shaker (Lab Rotator, Model #2314, Barnstead International, Dubuque, IA) at 40 rotations per minute continuously in 10 ml of ESC growth media without LIF^44^. Osteogenic differentiation of EBs was initiated by addition of 10 mM β-glycerophosphate (βGP, MP Biomedical, Solon, OH) to the media beginning at day 5 and continued until day 14.

### DBM Source and Inactivation

DBM derived from donated, human cadaveric tissue (Musculoskeletal Transplant Foundation, Edison, NJ) and previously validated to possess high osteoinductive potency was used as a positive control for *in vivo* ectopic bone formation studies. Inactive DBM was prepared by heating DBM from the same lot to 105 °C for 24 hours to denature osteoinductive factors present within the DBM and used as a negative control for *in vivo* experiments^49^.

### Preparation of EBM

EBM was derived from EBs at days 5, 10, and 14 of differentiation by lyophilization, as described previously^22^, from untreated EBs (-βGP) as well as osteogenic EBs (+βGP)^23^. Briefly, cell spheroids in each group were harvested (~2 x 10^3^ EBs per EBM sample), washed in PBS and resuspended in sterile, deionized water. After pelleting, tubes were frozen in water at -80 °C overnight and then lyophilized overnight to generate equal aliquots of devitalized material without any additional enzymatic or solvent treatments.

### Characterization of Devitalized Material

To assess gross morphology and physical characteristics, images of DBM and EBM were acquired using a dissecting stereomicroscope equipped with a digital camera (Nikon SMZ1500). Morphology was assessed microscopically by routine hematoxylin and eosin (H&E) staining (Leica AutoStainer XL) of formalin-fixed, paraffin-embedded sections. The presence of collagen within the devitalized materials was further evaluated by modified Mallory aniline blue (MMAB) staining^50^.

Growth factor retention within EBM and DBM was evaluated by enzyme linked immunosorbent assays (ELISA) using BMP-2 (PeproTech, Rocky Hill, NJ), BMP-4 and VEGF-A ELISA kits (R&D Systems, Minneapolis, MN;) per manufacturer’s instructions. EBM and DBM materials were solubilized at 3 mg/ml in Tissue Protein Extraction Reagent (T-PER; Pierce, Rockford, IL) for one hour at 4 °C by continuous rotational mixing. Experimental measurements were compared to a standard curve generated from known concentrations of individual proteins reconstituted in TPER buffer. Prior to implantation, EBM was screened to ensure low endotoxin levels (<0.5 EU/mL; QCL-1000, Lonza, Walkersville, MD).

### Preparation of Implants

For intramuscular implantation, DBM and EBM were loaded into UV-sterilized, size 5 gelatin capsules (Torpac, Fairfield, NJ). 15 mg of DBM (active or inactive) was loaded into each capsule for positive and negative controls, respectively. 7.5 mg of EBM (-βGP EBM, +βGP EBM) was mixed with 7.5 mg of inactive DBM before loading into individual capsules. Inactive DBM was used as a delivery vehicle for EBM implants due to its unique histological features and non-bioactive nature, enabling us to readily identify the sites of implanted EBM without interfering with the bioactivity of EBM.

### Implantation of DBM and EBM

All animal procedures were carried out in accordance with the approved guidelines for animal research at Georgia Institute of Technology. The experimental protocols were reviewed and approved by the Georgia Institute of Technology Institutional Animal Care and Use Committee. Sixteen male athymic Nu/Nu mice (Harlan, Indianapolis, IN) were randomly divided into four different groups: inactive DBM, -βGP EBM, +βGP EBM, active DBM. Different material formulations were implanted into the gastrocnemius with one implant per limb and the incision closed by wound clips. Each mouse received two of the same implant, to reduce systemic effects that influence response to an implant of a different type in the contralateral limb^51^, for a total of eight implants per experimental group. Mice were housed for 35 days under sterile conditions suitable for their immunocompromised state and provided food and water *ad libitum*.

### Evaluation of Mineralization – X-ray and Micro-computed Tomography (μCT)

Animals were euthanized at 35 days post-implantation by carbon dioxide asphyxiation for evaluation of mineralization and new bone formation. Hind limbs were removed and fixed in 10% neutral buffered formalin prior to X-ray examination to assess gross mineralization (Faxitron, Lincolnshire, IL). Mineralization at the site of implantation was further evaluated by μCT in air using a μCT 40 scanner (Scanco Medical, Brüttisellen, Switzerland) at 55 kVp, 145 μA, 200-ms integration time, and a voxel size of 30 μm in a 30 mm scanning tube. Evaluation of μCT scans used sigma, support and threshold values set at 3.3, 2, and 70, respectively. A minimum threshold (0.05%, 0.10 mm^3^) of absolute mineral volume per hindlimb volume (estimated from limb measurements) was established, above which limbs were scored as positive for mineralization by μCT.

### Histological Evaluation

Following analysis of mineralization, hind limbs were decalcified in 14% EDTA (Sigma, St. Louis, MO) in water (pH 7) for two weeks, with the progression of decalcification monitored daily by X-ray before proceeding to paraffin embedding and sectioning. New bone formation was evaluated histologically by semi-quantitative scoring and histomorphometric measurements, as previously described^4^. Three consecutive cross sections (5 μm) were collected at three different levels in the region of the implant along the longitudinal axis of the limb and stained with H&E (Leica AutoStainer XL). One complete section (of the nine total) per implant exhibiting the greatest amount of ossicle formation and residual DBM was selected for scoring and histomorphometric analysis. Due to the selection of the section containing residual DBM and the most new bone, the results were positively biased toward success for each implant type. Residual EBM and DBM within the implant site were evaluated by MMAB staining, as described previously, on sections adjacent to those chosen for scoring and histomorphometric evaluation. No residual EBM or DBM was included in the measurement of new bone.

For osteoinduction scoring, the entire section was evaluated by two independent blinded observers and graded according to a previously published semi-quantitative rating system^4^,^6^ (Supplementary Table 1). The same histological sections used for osteoinduction scoring were also evaluated by quantitative histomorphometric analysis using Metamorph™ software (v. 7.5, Molecular Devices, Sunnyvale, CA) to individually measure the total ossicle area (marrow space and associated new bone), as well as new bone and new bone marrow (distinct from DBM and limb bones).

### Statistical Analyses

Results are presented as mean and standard error, with n being the number of implant sites (n = 8), unless otherwise noted. For data not approximating a normal distribution, the data were normalized using a Box-Cox power transformation prior to statistical analysis^52^. For histomorphometry data that were not normally distributed even after transformation, Kruskal-Wallis non-parametric analysis of variance (ANOVA) and Tukey’s post-hoc tests were used to determine significant differences using MATLAB (Mathworks, Natick, MA). Otherwise, statistically significant differences were determined by one-way ANOVA followed by Tukey’s post-hoc test using Systat 12 (Chicago, IL). p < 0.05 was considered significant.

## Author Contributions

Conceived and designed the experiments: K.S., Z.S., B.B. and T.M.; performed the experiments: K.S., Z.S. and S.H.; analyzed the data: K.S., Z.S., Y.W.; contributed reagents and materials: ZS, BB. and TM; wrote the paper: K.S., Z.S., Y.W., B.B. and T.M.

## Additional Information

**How to cite this article**: Sutha, K. *et al*. Osteogenic Embryoid Body-Derived Material Induces Bone Formation *In Vivo*. *Sci. Rep.* **5**, 9960; doi: 10.1038/srep09960 (2015).

## Supplementary Material

Supplementary Information
