# Supplementary material for: Osteogenic Embryoid Body-Derived Material Induces Bone Formation In Vivo
Source: Sci Rep. 2015 May 11;5:9960. doi: 10.1038/srep09960 (PMC4426716; doi:10.1038/srep09960)
Supplement: Supplementary Information [file srep09960-s1.pdf]

Osteogenic Embryoid Body-Derived Material Induces Bone Formation *In Vivo*

Ken Sutha[1], Zvi Schwartz[2], Yun Wang [1], Sharon Hyzy[2],

Barbara D. Boyan[1,2], Todd C. McDevitt[1,3*]

[1]Wallace H. Coulter Department of Biomedical Engineering, Georgia Institute of Technology & Emory University, 313 Ferst Drive, Atlanta GA, 30332-0535, USA

[2]Department of Biomedical Engineering, School of Engineering, Virginia Commonwealth University, Richmond, VA, 23284-3068, USA

[3]Parker H. Petit Institute for Bioengineering and Bioscience, Georgia Institute of Technology, 315 Ferst Drive, Atlanta GA, 30332-0532, USA

***Corresponding Author: Todd C. McDevitt, Ph.D.**

315 Ferst Drive

Atlanta GA, 30332-0532

Phone: 404.385.6647

Fax: 404.894.4243

Email: todd.mcdevitt@bme.gatech.edu

Supplementary Table

Supplementary Table 1. Osteoinduction Scoring

Osteoinduction Score	Observation Within Entire Limb Section
0	No residual DBM or EBM
1	Residual DBM or EBM but no new bone formation
2	One single ossicle
3	Ossicle formation at multiple sites

Supplementary figures

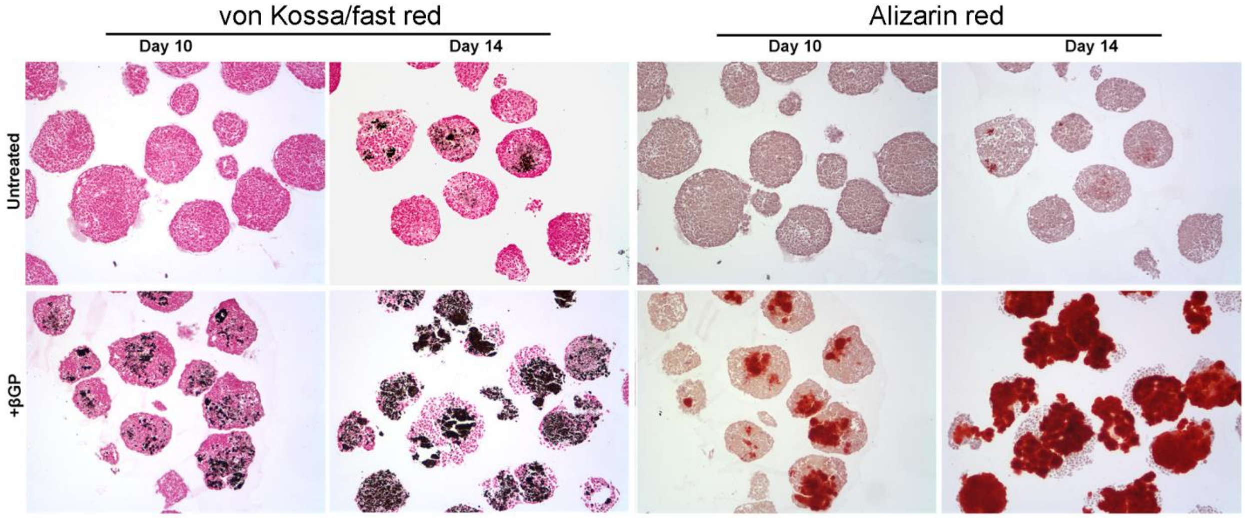

Supplementary Fig. S1 Mineralization in EBs cultured under different conditions. EBs differentiated in the absence or presence of βGP (10 μM) beginning at day 5 of EB differentiation. The mineralization of EBs was evaluated using both von Kossa and Alizarin Red staining (scale bar = 400 μm).

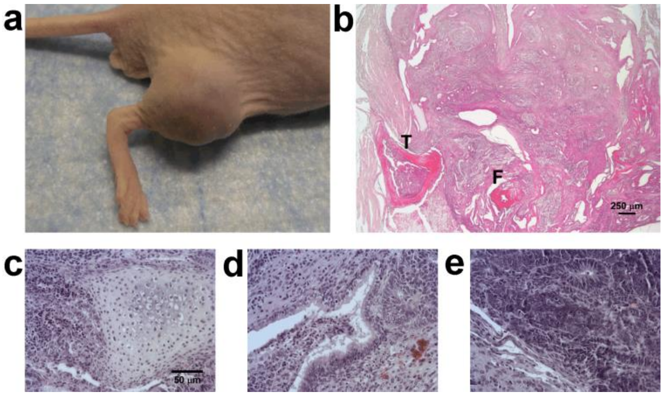

Supplementary Fig. S2 Teratoma formation 28 days post-implantation of viable day 10 EBs. Viable day 10 EBs, with and without βGP treatment, were implanted into mouse hindlimbs. Large masses in the hindlimbs were apparent by 28 days post-implantation (a), and when evaluated histologically, the masses were confirmed to be teratomas (b) (T: Tibia, F: Fibula,

scale bar = 250 μm), which comprised of cells from all three germ lineages: mesoderm (c), endoderm (d), and ectoderm (e), scale bar = 50 μm.

Supplementary methods

Histology analysis of mineralization in ESC aggregates

Paraffin-embedded ESC aggregates were sectioned at a thickness of 5 μm and subjected to routine von Kossa/fast red and alizarin red staining to visualize mineralization within aggregates. Stained sections were imaged using a Nikon Eclipse 80i equipped with a SpotFlex digital camera (Diagnostic Instruments, Sterling Heights, MI).

Teratoma formation assay

All studies were performed with a Georgia Institute of Technology Institutional Animal Care and Use Committee approved protocol. Male SCID mice (8-week-old) were used for all experiments. D10 ESC aggregates were harvested after *in vitro* culture and resuspended in saline and injected into the hindlimbs of the mice. After 28 days of implantation, the tissues were harvested for histological analysis. Paraffin-embedded teratoma tissues were sectioned at a thickness of 5 μm and subjected to routine hematoxylin and eosin staining to examine the tissue formation.
